# Potential Regulation of *ARID1A* by miR-129-5p and miR-3613-3p and Their Prognostic Value in Gastric Cancer

**DOI:** 10.3390/ijms26010305

**Published:** 2025-01-01

**Authors:** Irina V. Bure, Ekaterina A. Vetchinkina, Alexey I. Kalinkin, Ekaterina B. Kuznetsova, Artem D. Molchanov, Alevtina E. Kiseleva, Ekaterina A. Alekseeva, Neonila V. Gorokhovets, Ivan V. Rodionov, Marina V. Nemtsova

**Affiliations:** 1 I.M. Sechenov First Moscow State Medical University (Sechenov University), 119991 Moscow, Russia; 2 Research Institute of Molecular and Personalized Medicine, Russian Medical Academy of Continuous Professional Education, 125993 Moscow, Russia; 3 Laboratory of Epigenetics, Research Centre for Medical Genetics, 115522 Moscow, Russia

**Keywords:** *ARID1A*, miR-129-5p, miR-3613-3p, microRNA, gastric cancer, cancer cell lines, epigenetics, biomarker

## Abstract

Gastric cancer (GC) remains the most common malignant tumor of the gastrointestinal tract and one of the leading causes of cancer-related deaths worldwide. Non-coding RNAs (ncRNAs), including microRNAs (miRNAs), are involved in the pathogenesis and progression of GC and, therefore, may be potential diagnostic and prognostic biomarkers. Our work was aimed at investigating the predicted regulation of *ARID1A* by miR-129-5p and miR-3613-3p and the clinical value of their aberrant expression in GC. The study included tumor and adjacent non-tumor tissues from 110 GC patients, 38 sectional normal gastric tissue samples, as well as 65 plasma samples of GC patients and 49 plasma samples of healthy donors. Expression levels of *ARID1A* and both miRNAs were quantified by reverse transcription-polymerase chain reaction (RT-PCR). We have identified significant associations of their expression with the clinical and pathological characteristics of GC patients both in tissues and plasma. To validate predicted target pairs miR-129-5p/*ARID1A* and miR-3613-3p/*ARID1A*, in vitro experiments on cancer cell lines were conducted. The obtained results suggest a complex role of ARID1A, miR-129-5p and miR-3613-3p in GC and potential regulation of *ARID1A* expression by both miRNAs.

## 1. Introduction

Gastric cancer (GC) is one of the most common malignant tumors worldwide. It is often diagnosed at late stages characterized by tumor invasion and distant metastases, which lead to unfavorable prognosis for patients. Therefore, search for novel non-invasive biomarkers remains important for early diagnostics, postoperative monitoring, and effective treatment [1]. MicroRNAs (miRNAs) circulating in the plasma or serum of patients are considered as such candidate biomarkers [2,3,4].

MiRNAs epigenetically regulate the expression of target genes by binding to the complementary sequence on the 3′-untranslated region (3′-UTR) of mRNA, thus causing its degradation or suppressing translation [5]. MiRNAs are involved in the development and progression of many diseases, including malignant neoplasms [6,7,8,9]. Aberrant miRNA expression patterns are associated with clinical characteristics and molecular classification of different types of cancer, which make them potential diagnostic and prognostic markers [10].

Aberrant expression of miR-129-5p was described in many types of tumors, such as breast cancer [11], colon cancer [12], osteosarcoma [13], lung cancer [14], and GC [15,16]. MiR-3613-3p remains less investigated; however, its differential expression profile was confirmed in plasma and tissues of patients with colorectal cancer and GC [17,18]. MiR-3613-3p is often deleted in breast cancer [19] and affects cell proliferation and cell cycle in hepatocellular carcinoma [20]. Differential expression of miR-3613-3p was observed in plasma from children with retinoblastoma [21] and in neuroblastoma cell lines [22]. Differential miR-3613-3p expression is associated with somatic mutations in the *TP53* in colorectal cancer [23] and with metastasis to regional lymph nodes in endometrial cancer [24].

The choice of miR-129-5p miR-3613-3p for our study was based on their analysis by computer algorithms TargetScanHuman 7.1. [25] and mirDIP [26] that use comparison of conservative binding sites of transcripts. The algorithms predicted *ARID1A* as a potential target for both miR-129-5p and miR-3613-3p, which makes them interesting for further investigation.

*ARID1A* (AT-rich interactive domain 1A) is a known tumor suppressor gene [27]. Being one of the subunits of the SWI/SNF chromatin remodeling complex, it plays an important role in proliferation, invasion, cell cycle regulation, metastasis, DNA repair, and apoptosis [28,29]. *ARID1A* was described as often mutated in various types of tumors, such as clear cell ovarian cancer (45%), endometrial cancer (37%), GC (20–30%), bladder cancer (20%), hepatocellular cancer (14%), melanomas (12%), colorectal cancer (9%), lung cancer (8%), pancreatic cancer (4%), and breast cancer (3%) [30]. Clinically, loss of *ARID1A* expression correlates with larger tumor sizes, deeper invasion, lymph node metastases and poor prognosis in GC patients [31].

Taking into account the important role of these transcripts in GC, in our work, we evaluated the expression level of miR-129-5p and miR-3613-3p, as well as *ARID1A* in tissues and plasma of GC patients and healthy donors. To determine their potential as biomarkers for GC, we investigated their associations with clinical and pathological characteristics of tumor growth and microsatellite instability (MSI). In addition, since *ARID1A* was predicted as a potential target gene for miR-129-5p and miR-3613-3p, we conducted experiments on cancer cell lines to validate this regulation in vitro.

## 2. Results

### 2.1. ARID1A and miR-129-5p Are Differentially Expressed in Tissues from GC Patients and Sectional Normal Gastric Tissues

**Figure 1:**
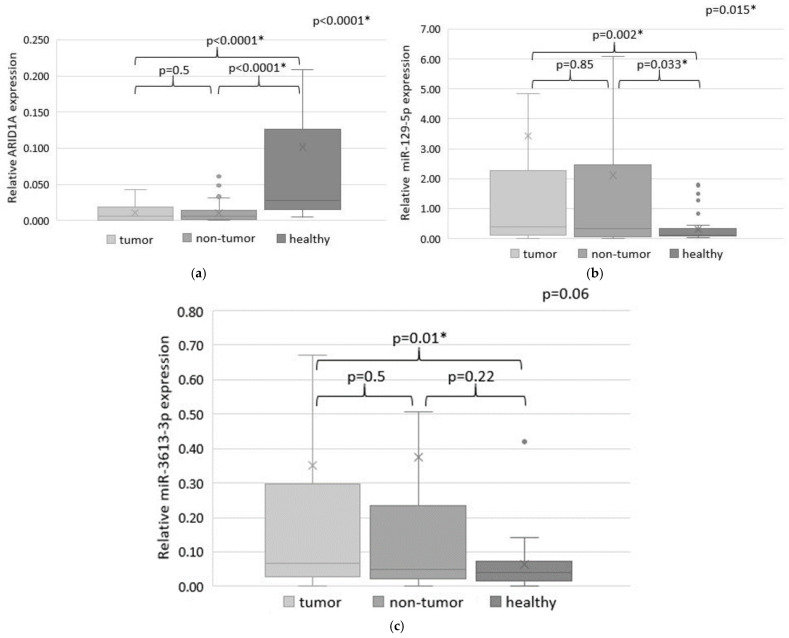
The expression levels of *ARID1A* (**a**), miR-129-5p (**b**) and miR-3613-3p (**c**) in tumor and adjacent non-tumor tissues of GC patients and in normal gastric tissue samples (colored in different shades of grey). Statistically significant *p*-values are indicated with an asterisk.

The expression levels of *ARID1A* and miRNAs were investigated in 110 paired samples of tumor and adjacent non-tumor tissues of GC patients, as well as in 38 sectional normal gastric tissue samples. Quantitative analysis revealed statistically significant differences in *ARID1A* expression in tumor and non-tumor tissues compared to sectional gastric tissues of the healthy control group (*p* < 0.0001) (Figure 1a). *ARID1A* was upregulated in 51 (46%) tumor samples and downregulated in 59 (54%) tumor samples. However, the average level of ARID1A expression in tumor tissues compared with adjacent non-tumor tissue of GC patients did not differ statistically (*p* = 0.5).

MiR-129-5p expression was also different in tumor and non-tumor tissues of GC patients compared to gastric tissues of a healthy control group (*p* = 0.002 and *p* = 0.0033, respectively) (Figure 1b). MiR-129-5p expression was increased in 53 (48%) and decreased in 57 (52%) tumor tissue samples compared to the non-tumor tissue of GC patients, but the average miR-129-5p expression levels were not statistically significant (*p* = 0.85).

A comparison of miR-3613-3p expression in tissues of GC patients and sectional normal gastric samples of a healthy control group showed statistically significant differences only for tumor tissue (*p* = 0.01) (Figure 1b). An increased miR-3613-3p expression in the tumor compared to the non-tumor tissue was observed in 58 (53%) samples, and decreased miR-3613-3p expression was in 52 (47%) samples. However, no statistically significant differences were found when comparing expression in tumor and adjacent non-tumor tissue.

### 2.2. Predicted Target Pairs miR-129-5p/ARID1A and miR-3613-3p/ARID1A Demonstrate Negative Correlation in Tumor and Adjacent Non-Tumor Tissues of GC Patients

**Figure 2:**
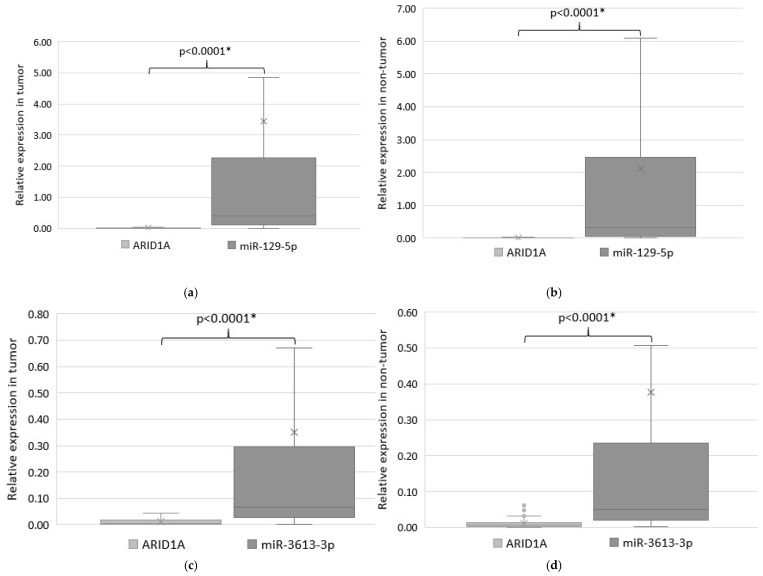
Relative expression of *ARID1A* and miR-129-5p (colored in different shades of grey) in tumor tissues (**a**) and in the adjacent non-tumor tissues (**b**) of GC patients; relative expression of *ARID1A* and miR-3613-3p in tumor tissues (**c**) and the adjacent non-tumor tissues (**b**) of GC patients, and in normal gastric tissue samples (**d**). Statistically significant *p*-values are indicated with an asterisk.

As *ARID1A* was predicted to be a potential target of miRNAs miR-129-5p and miR-3613-3p, we analyzed their expression patterns in tissues of GC patients. Significant inverse correlations were confirmed in both target pairs in tumor (*p* < 0.0001) (Figure 2a,c), as well as in adjacent non-tumor tissues (*p* < 0.0001) (Figure 2b,d).

**Figure 3:**
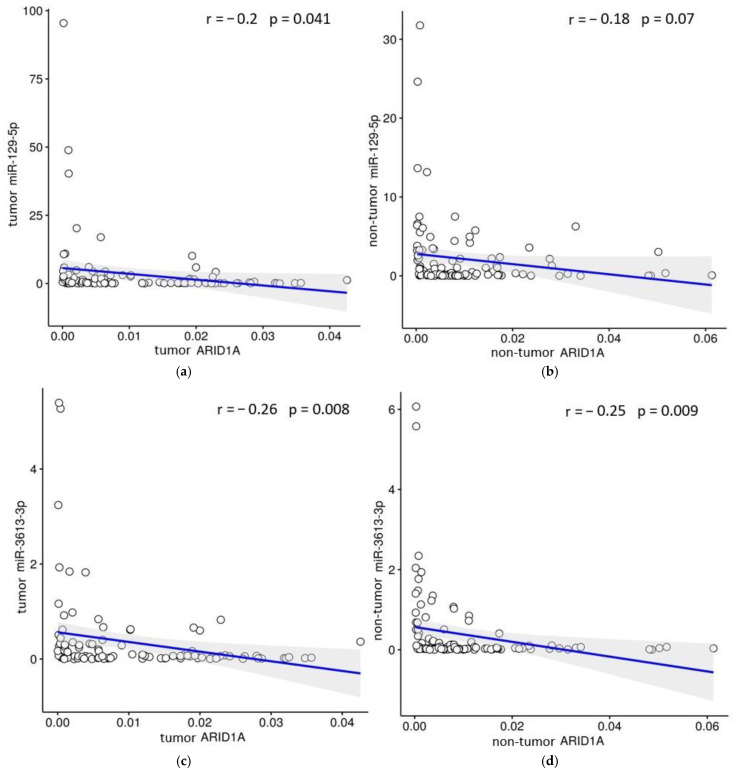
Linear regression showing the negative correlation of expression levels in pars *ARID1A*/miR-129-5p and *ARID1A*/miR-3613-3p in tumor (**a**,**c**) and non-tumor tissue (**b**,**d**) of GC patients.

The Spearman’s correlation analysis revealed only weak negative relevance between *ARID1A* and miR-129-5p expression in tumor r = −0.2 (*p* = 0.04) (Figure 3a) and non-tumor tissues r = −0.18 (*p* = 0.07) (Figure 3b) of GC patients. When evaluating the potential regulatory pair *ARID1A*/miR-3613-3p, we also found a statistically significant weak negative correlation between expression in tumor r = −0.26 (*p* = 0.008) (Figure 3c) and in non-tumor tissues r = −0.25 (*p* = 0.009) (Figure 3d). Therefore, the analysis may also be related to determined differences in expression levels of *ARID1A* and miRNAs in tissues of GC patients.

### 2.3. Overexpression of miR-129-5p or miR-3613-3p Significantly Reduce ARID1A mRNA Abundance in Cancer Cells

**Figure 4:**
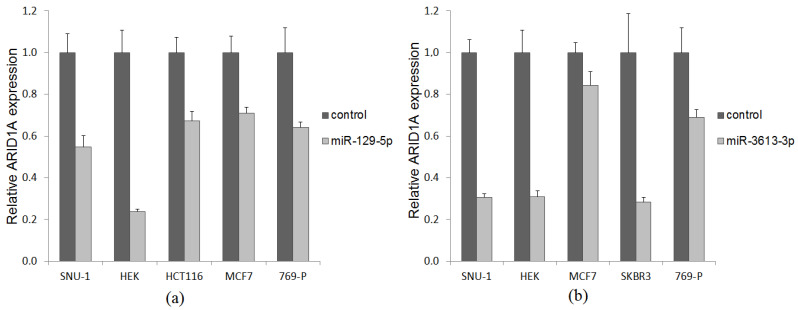
*ARID1A* mRNA abundance in cancer cells transfected with miR-129-5p (**a**) and miR-3613-3p (**b**) expression plasmid (light grey bar) and empty vector (dark grey bar).

To experimentally prove an inverse correlation in predicted target pairs, miR-129-5p and miR-3613-3p were ectopically expressed in cell lines of different cancer types, including SNU-1 (gastric cancer), HCT116 (colon cancer), MCF-7 and SKBR3 (breast cancer), 769-P (renal cell carcinoma) and A549 (lung cancer) followed by quantification of *ARID1A* expression. For all of them, the ectopic expression of respective miRNA was at least threefold higher compared to the endogenous expression in untransfected cell lines. MiR-129-5p induced a reduction of *ARID1A* mRNA quantity in cell lines SNU-1 (45%), HEK (76%), HCT116 (33%), MCF-7 (29%), and 769-P (36%) (Figure 4a). Overexpression of miR-3613-3p led to a decrease of *ARID1A* expression in cell lines SNU-1 (68%), HEK (69%), MCF-7 (16%), SCBR3 (72%) и 769-P (31%) SNU-1 cells.

### 2.4. Expression Levels of ARID1A, miR-129-5p and miR-3613-3p Are Associated with Clinical and Pathological Characteristics of GC Patients

To determine the significant factors related to the clinical course of GC, we analyzed potential associations between the expression of *ARID1A*, miR-129-5p and miR-3613-3p in tumor tissues of GC patients and their clinical and pathological characteristics.

It was found that increased *ARID1A* expression correlates with the diffuse type of GC according to Lauren classification (*p* = 0.001), the presence of the signet ring cells (*p* = 0.044) and mutations in the *CDH1* gene (*p* = 0.04). Alternatively, an increased miR-3613-3p expression was associated with the intestinal type of GC (*p* = 0.011). Both *ARID1A* and miR-3613-3p were associated with the primary tumor size (T in the TNM classification). Moreover, T3 + T4 group was characterized by *ARID1A* downregulation (*p* = 0.037) and miR-3613-3p upregulation (*p* = 0.011). The expression levels of *ARID1A* and both miR-129-5p and miR-3613-3p were also correlated with the overall survival status (*p* = 0.038, *p* = 0.017 and *p* = 0.027, respectively).

When comparing with the other characteristics, no significant correlations were found. The results of all estimated correlations are presented in Table 1.

A similar analysis of associations between the expression of *ARID1A*, miR-129-5p and miR-3613-3p and their clinical and pathological characteristics was also performed in non-tumor tissues of the same GC patients. Increased *ARID1A* expression was found to correlate with the diffuse type of GC according to Lauren classification (*p* = 0.002) and mutations in the *TP53* (*p* = 0.038). The differential expression of miR-129-5p and miR-3613-3p was associated with the overall survival status of patients (*p* = 0.035 and *p* = 0.024, respectively). Their increased expression was associated with microsatellite instability of H&L (*p* = 0.023 and *p* = 0.018, respectively). Correlations were also found between miR-3613-3p expression and the primary tumor size T and age of GC patients. The increased miR-3613-3p expression was associated with the T3 + T4 group (*p* = 0.021) and older age (*p* = 0.011). No significant correlations were found for other characteristics. The results of all estimated correlations are presented in Table 2.

### 2.5. Associations of ARID1A, miR-129-5p and miR-3613-3p Expression Levels with Overall Survival of GC Patients Were Estimated

**Figure 5:**
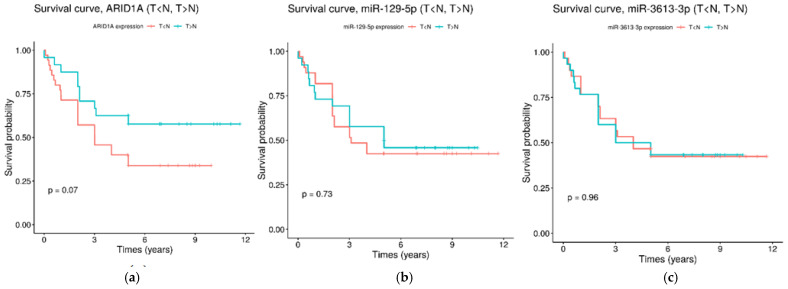
Kaplan–Meier survival curves for the overall survival of GC patients, depending on the expression of ARID1A (**a**), miR-129-5p (**b**) and miR-3613-3p (**c**). Red survival curves are for the group of patents with upregulated in the tumor tissue transcripts (T > N) and green survival curves are for the group of patents with upregulated in the adjacent non-tumor tissue transcripts (T < N).

Using the Kaplan–Meier method, we estimated associations between the expression of *ARID1A*, miR-129-5p and miR-3613-3p and the overall survival of GC patients. The patients were divided into two groups depending on the transcript expression levels: upregulated in the tumor tissue (T > N) and upregulated in the adjacent non-tumor tissue (T < N). However, no significant associations with the overall survival of GC patients were found for all transcripts (Figure 5).

### 2.6. MiR-129-5p Abundance Is Significantly Different in Plasma of GC Patients and Healthy Donors and Correlates with Clinical and Pathological Characteristics of GC Patients

**Figure 6:**
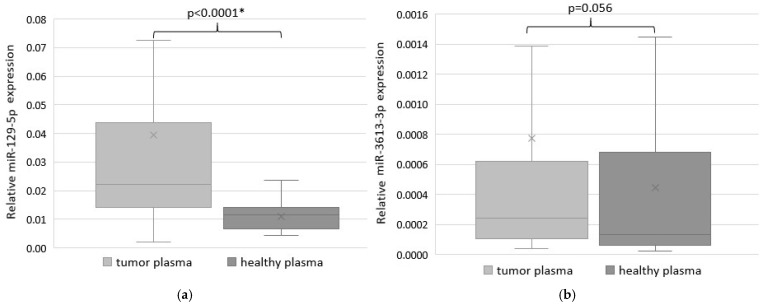
The expression level of miR-129-5p (**a**) and miR-3613-3p (**b**) in the plasma of GC patients and healthy donors. Statistically significant *p*-values are indicated with an asterisk.

To estimate the diagnostic potential of miR-129-5p and miR-3613-3p, their expressions were quantified in 65 plasma samples of GC patients and 40 plasma samples of healthy donors as control. MiR-129-5p abundance was significantly higher in the plasma of GC patients compared to control (*p* < 0.0001) (Figure 6a). No significant difference was found for miR-3613-3p (*p* = 0.056) (Figure 6b).

To determine factors related to the clinical course of GC, we analyzed associations between miR-129-5p and miR-3613-3p abundance in plasma and clinical characteristics of tumor growth in GC patients. It was found that increased miR-129-5p expression was significantly associated with T3 + T4 group according to the TNM classification (*p* = 0.005), the development of metastases to regional lymph nodes (N1–3) (*p* = 0.005) and development of distant metastases (*p* = 0.004), as well as late stages of GC (stage III + IV) (*p* = 0.006).

No significant associations were found for miR-3613-3p. The results of all estimated correlations are presented in Table 3.

### 2.7. Diagnostic Value of miR-129-5p and miR-3613-3p Was Estimated by the ROC-Curves

**Figure 7:**
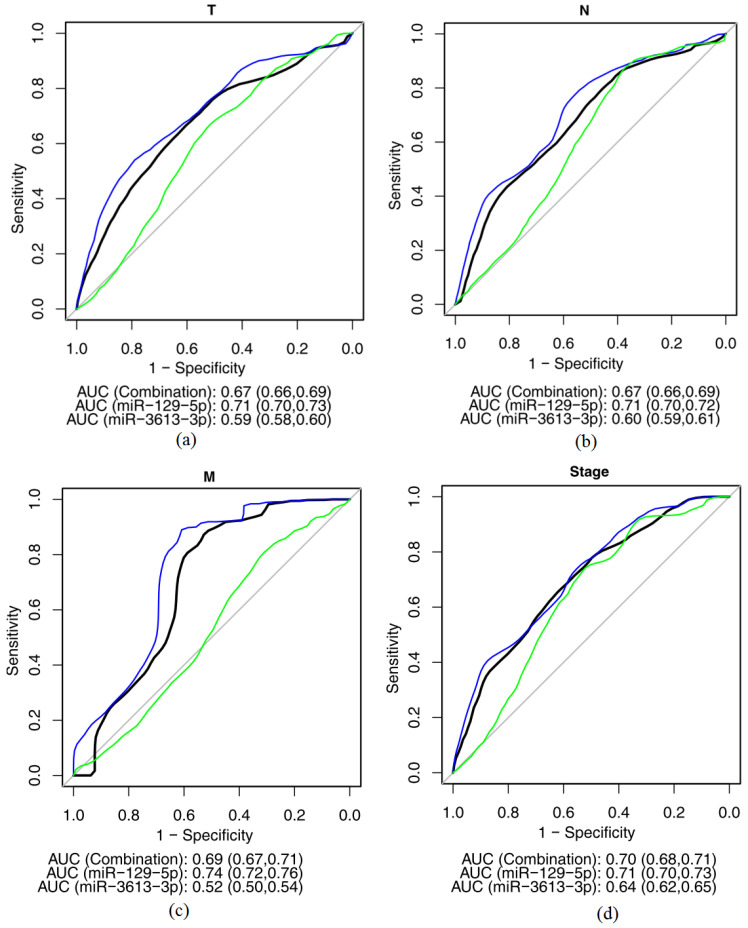
Associations of miR-129-5p and miR-3613-3p abundance in plasma of GC patients with the primary tumor size (**a**), metastases to regional lymph nodes (**b**), distant metastases (**c**), the clinical stage of GC (**d**). ROC-curves: blue—miR-129-5p, green—miR-3613-3p, black—a combination of both microRNAs.

To estimate the potential clinical relevance of miR-129-5p and miR-3613-3p, a receiver operating characteristic (ROC-curves) was used. MiRNAs abundance in the plasma of GC patients was compared with their clinical and pathological characteristics with consequent one-dimensional logistic regression analysis to calculate the area under the ROC-curve (AUC) with a statistical significance level of more than 0.5. Acceptable values of AUC (>0.7) were demonstrated for associations of miR-129-5p abundance with the primary tumor size, metastases to regional lymph nodes, distant metastases and clinical stages of GC (Figure 7). The combination of miR-129-5p and miR-3613-3p did not improve the prognostic value of the system (Table 4).

## 3. Discussion

In recent years, many ncRNAs have been identified as important participants in tumorigenesis. MiRNAs regulate many genes, including oncogenes and tumor-suppressors. Being aberrantly expressed, such miRNAs affect numerous signaling pathways and thus can lead to tumor initiation and progression [32,33]. Given the significant role of miRNAs in oncogenesis and their disease-specific expression patterns, they could be considered as potential therapeutic targets and novel biomarkers [34]. In our work, we quantified the expression of miR-129-5p and miR-3613-3p in tissues and plasma of GC patients, as well as in normal gastric tissue samples and plasma from healthy donors, to investigate their clinical value.

We revealed no differences between miR-129-5p expression levels in tumor and adjacent non-tumor tissues of GC patients (*p* = 0.85). However, miR-129-5p expression in both tumor and non-tumor tissues of GC patients was significantly different compared to the sectional samples of normal gastric tissue (*p* = 0.002 and *p* = 0.033, respectively). Such differences confirm that miR-129-5p expression depends on tumor lesions and can be deregulated in both tumor and non-tumor tissues of GC patients. The miR-3613-3p expression was also not different when we compared the tumor and non-tumor tissues of GC patients (*p* = 0.5) but was significantly different in tumor tissues and gastric normal tissues of the control group (*p* = 0.01). The aberrant expression pattern of both miRNAs in histologically normal non-tumor tissues of GC patients could be related to the field of cancerization. Therefore, despite the absence of tumor cells, the non-tumor tissues were affected by the growing tumor and are characterized by genetic and epigenetic alterations.

It was already described that increased miR-129-5p expression suppresses GC progression by interacting with its target genes, such as *HMGB1* [35], *SPOCK1* [36], *COL1A1* [16], *HOXC10* [37], and *IL-8* [38]. Previous investigations have also demonstrated that ectopic miR-129-5p expression suppresses the proliferation and invasiveness of GC cells by inducing G1 phase arrest in vitro and inhibits tumor growth in vivo [37]. Our results were different from those published earlier: miR-129-5p expression level was significantly lower in the tissues of GC patients compared to the healthy controls. It may be related to the heterogeneity of our sample set, where miR-129-5p was both downregulated and upregulated in different samples. Similar ambiguous changes in miR-129-5p expression have already been reported in hepatocellular carcinoma [39].

The analysis of plasma samples from GC patients and healthy donors also revealed correlations of miR-129-5p expression with clinical and pathological characteristics of GC patients. Thus, increased miR-129-5p expression was significantly associated with T3 + T4 group according to the TNM classification (*p* = 0.005) and later stages of the disease (stage III + IV) (*p* = 0.006), as well as with metastases to regional lymph nodes (N1–3) (*p* = 0.005) and distant M1 metastases (*p* = 0.004).

In our study, the average miR-3613-3p expression level was increased in the tumor tissue of GC patients when compared to the normal gastric tissues of a healthy control group (*p* = 0.01). In plasma samples of GC patients, miR-3613-3p was also upregulated but not statistically significant. Our data are in concordance with results obtained by Bibi et al. In their study of GC patients from the Saudi Arabian population in the early and late stages of cancer, miR-3613-3p expression was also increased compared to the healthy gastric samples [18]. In addition, increased miR-3613-3p expression was shown to promote proliferation and metastasis in breast cancer cells by targeting *SOCS2* [40].

We have shown an association of miR-3613-3p expression in the tumor tissue of GC patients with the status of overall survival (*p* = 0.027), the Lauren classification (*p* = 0.011) and the primary tumor size T (*p* = 0.011). Statistically significant differences in miR-3613-3p expression levels were also found in the adjacent non-tumor tissue of GC patients. There are associations with the age group (*p* = 0.011), overall survival (*p* = 0.024), the primary tumor size T (*p* = 0.021) and the presence or absence of MSI (*p* = 0.018). Moreover, according to these clinical and pathological characteristics, low expression of miR-3613-3p was associated with a worse prognosis. Differential miR-3613-3p expression was observed in tissues and plasma of patients with lung adenocarcinoma and was associated with TNM stages [41]. However, in the plasma of GC patients, we could not find any significant associations of miR-3613-3p expression when compared with various clinical and pathological characteristics. The small amount of published data on miR-3613-3p suggests that its role in GC remains unclear.

The high frequency of *ARID1A* mutation in cancer highlights its importance in oncogenesis. Mutations in *ARID1A* are usually inactivating (for example, nonsense mutations, reading frame shift mutations, mutations of one allele with loss of heterozygosity of another) and lead to loss of gene function and prevention of corresponding protein synthesis [42] and are associated with an increased risk of GC [43]. In normal cells, accumulation of the produced ARID1A protein is detected during the G1 phase of the cell cycle, and a strong decrease occurs during the G1–S phase [44]. The loss of ARID1A protein was heterogeneous in GC tumor tissue, thus suggesting that *ARID1A* mutations may be a later event of gastric carcinogenesis [45]. The recent review discussed the dual role of *ARID1A* as a suppressor gene and as an initiator of tumor progression [44,46].

In our study, *ARID1A* expression was significantly lower in tumor and adjacent non-tumor tissues compared to sectional normal gastric samples (*p* < 0.0001). However, no significant differences were found between the tumor and non-tumor tissues of GC patients (*p* = 0.5). A decreased *ARID1A* expression was already reported in GC tumor tissues, and it also was not statistically significant compared to the adjacent non-tumor tissue [47]. Wang et al. showed a statistically significant decrease in *ARID1A* expression in tumor tissues compared to non-tumor tissues of GC patients [48]. A decrease or loss of the ARID1A protein was observed in about 22% of GC cases [49,50].

In hepatocellular carcinoma, *ARID1A* demonstrated a higher expression in primary tumors than in metastatic tumors, which suggests that *ARID1A* expression may be decreased after the initiation of oncogenesis [51]. We have shown that a low *ARID1A* expression in the tumor tissue of GC patients is significantly associated with a large primary tumor size (T3 + T4) (*p* = 0.037). Previously published studies also confirmed that reduced *ARID1A* expression is often associated with late stages of oncogenesis [48,52,53]. The decreased level of *ARID1A* protein in GC patients was associated with an increase in tumor invasion and, thus, with the progression of the disease rather than the early stages [54]. This indicates the role of *ARID1A* expression in primary cancer in various types of tumors.

The analysis of the overall survival of GC patients revealed that *ARID1A* expression in tumor tissues was significantly higher in patients who survived at the time of the study (*p* = 0.038). However, no significant differences were found when we compared survival using the Kaplan–Meier method. It has previously been shown that the downregulation of *ARID1A* was correlated with a poor prognosis for GC patients [55,56]. Low *ARID1A* expression was also associated with the survival status of GC patients [57,58,59]. In our study, *ARID1A* expression was correlated with the type of tumor according to Lauren classification in both tumors (*p* = 0.001) and non-tumor tissue (*p* = 0.002), which is in concordance with the other investigation [45].

*ARID1A* expression was also associated with somatic mutations in tumor driver genes. Thus, its expression in tumor tissue correlated with mutations in *CDH1* (*p* = 0.04), whereas its expression in non-tumor tissues of GC patients was correlated with mutations in *TP53* (*p* = 0.038). In a study conducted on cell cultures, it was shown that knockdown of *ARID1A* led to decreased *CDH1* expression [60]. *TP53* and *ARID1A* are known to be frequently mutated in different tumor cells but rarely in the same primary tumor [61,62]. In our study, *ARID1A* expression in non-tumor tissue of GC patients was significantly higher in samples with mutated *TP53*. *ARID1A* expression in tumor tissue of GC patients was also higher in samples with mutated *TP53*. However, this difference was not statistically significant (*p* = 0.2). It was suggested that the deregulation of *ARID1A* and *TP53* may affect independent tumor progression pathways [52].

Currently, a number of studies describe the direct regulation of *ARID1A* by miRNAs in different types of tumors. For example, miR-7641 promotes cell proliferation and colony formation [63], and miR-223-3p promotes cell invasion and proliferation [64] in GC by directly targeting *ARID1A*. MiR-221 and miR-222 simultaneously target *ARID1A* and enhance the proliferation and invasion of cervical cancer cells [65]. MiR-144-3p downregulates *ARID1A* and thus promotes cell proliferation, metastasis, and Sunitinib resistance in clear cell renal cell carcinoma [66], whereas miR-31 functions as an oncomir for *ARID1A* in cervical cancer [67].

The algorithms TargetScanHuman 7.1 and mirDIP predicted miR-129-5p and miR-3613-3p as potential regulators of the *ARID1A* based on the complementarity of their binding sites. We found an inverse correlation between the expression levels in tumor and adjacent non-tumor tissues of GC patients for both pairs (*p* < 0.0001). Evaluation of the correlation between transcript expression levels in potential regulatory pairs by Spearman’s rank correlation coefficient showed only a weak negative linear relationship between the expression levels of *ARID1A* and miR-129-5p in tumor r = −0.2 (*p* = 0.04) and a weak negative linear relationship between the expression of *ARID1A* and miR-3613-3p in tumor r = −0.26 (*p* = 0.008) and adjacent non-tumor tissues r = −0.25 (*p* = 0.009), which may also indicate the inverse correlations in the pairs miR-129-5p/*ARID1A* and miR-3613-3p/*ARID1A*. To further address their potential regulation, both mRNA/miRNA pairs were validated in vitro on a panel of 6 cancer cell lines. Ectopic expression of each of the investigated miRNAs resulted in a significant decrease in *ARID1A* expression in almost all cell lines by 29–76%.

Therefore, we can suggest that *ARID1A*, miR-129-5p and miR-3613-3p are aberrantly expressed in gastric cancer and involved in its tumorigenesis, and additional studies are needed to shed light on their functions and prognostic value.

## 4. Materials and Methods

### 4.1. Patients

The study included 110 paired samples of GC tumor material and morphologically normal adjacent non-tumor tissue and the independent set of 65 plasma samples of GC patients. All patients were diagnosed and undergone surgical treatment at the N.N. Burdenko Elective Surgery Clinic of the I.M. Sechenov First Moscow State Medical University. GC was confirmed by morphological examination of the surgical material. The clinical and pathological characteristics of patients are presented in Table 5.

Additionally, 38 sectional normal gastric tissue samples from people who died for reasons unrelated to cancer and 49 plasma samples from healthy donors with no history of cancer were included in the investigation as controls.

All subjects gave their informed consent for inclusion before they participated in the study. The study was conducted in accordance with the Declaration of Helsinki and the protocol №04-19 approved by the Ethics Committee of Sechenov First Moscow State Medical University (Sechenov University) on 06 March 2019.

### 4.2. Tissue and Plasma Samples

The tissue samples were frozen directly after surgery and stored at −80 °C. Blood samples from each patient and healthy donor were collected into K3EDTA tubes. Plasma was obtained by centrifugation and frozen at −80 °C. The material was annotated with an indication of localization, clinical stage, classification according to Lauren, the presence or absence of signet ring cells, metastases to regional lymph nodes and distant metastases, as well as according to the TNM classification.

### 4.3. Cell Lines

The cell lines were purchased from the American Type Culture Collection. The cell lines SNU-1 и 769-P were grown in RPMI 1640 medium, supplemented with 10% fetal bovine serum (*v/v*) and 1% of antibiotic mixture penicillin–streptomycin (*v/v*) (all from Gibco, Waltham, MA, USA). The HCT116, MCF7, SKBR3 and A549 were grown in DMEM medium supplemented with 10% fetal bovine serum (*v/v*) and 1% of antibiotic mixture penicillin-streptomycin (*v/v*) (Gibco, USA). All cell lines were incubated at 37 °C in a humidified atmosphere containing 5% CO2. All tested cell lines were authenticated by STR DNA Profiling Analysis (GORDIZ, Moscow, Russia).

### 4.4. RNA Extraction and Reverse Transcription-Polymerase Chain Reaction (RT-PCR)

Total RNA was extracted from samples by using Trizol (Life Technologies, Carlsbad, CA, USA) and the miRNeasy Mini Kit (Qiagen, Hilden, Germany) according to the protocol of manufacturers with small modifications. The NanoDrop 2000 micro-volume spectrophotometer (Thermo Fisher Scientific, New York, NY, USA) was used to estimate the concentration and purity of the obtained RNA.

For each sample, cDNA was synthesized from 300 ng of total RNA using MiScript II RT Kit (Qiagen) under the recommended protocol. Real-time PCR was performed on the CFX96 Real-Time PCR Detection System (Bio-Rad, Hercules, CA, USA) in three repetitions for each transcript and control, using the MiScript SYBR Green PCR Kit (Qiagen) according to the manufacturer’s protocol. Housekeeping gene *GAPDH* (Glyceraldehyde 3-phosphate dehydrogenase) and snRNA RNU6B were used as controls for *ARID1A* and miRNAs, respectively, when the expression in tissue samples was analyzed. The primer sequences are listed in Table 6. Exogenous control cel-miR-39-3p was used as a control for miRNAs in the plasma sample. Presynthesized miScript Primer Assay (Qiagen) primer was used for cel-miR-39-3p. Data of the ncRNAs expression were normalized and analyzed using the ΔΔCt method. The obtained Ct values were normalized and analyzed using the 2-ΔCt method and were presented as relative expression units (REU) [68].

### 4.5. MSI Analysis

The status of microsatellite instability (MSI) was determined by using fragment analysis on an ABI PRISM 3500 (8 capillaries; 50 cm; Applied Biosystems, Waltham, MA, USA) with five mononucleotide markers (NR21, NR24, NR27, BAT25, and BAT26) (Table 7) [69].

DNA fragments were amplified by PCR according to the previously described protocol [70].

### 4.6. Screening for CDH1 and TP53 Mutations by NGS

Genomic DNA was isolated from formalin-fixed paraffin-embedded (FFPE) samples by a QIAamp DNA FFPE Tissue kit, as recommended by Qiagen (Germany). Deep sequencing was performed using the Ion Torrent platform (Life Technologies) following the established protocol [71].

We utilized our original, customized panel, comprised of six hereditary GC-related genes (HGC panel) [70]. An HGC panel with 218 primer pairs was designed to amplify all coding regions, noncoding regions of the terminal exons, and putative splice site gene regions for six human genes: *BMPR1A*, *SMAD4*, *CDH1*, *TP53*, *STK11*, and *PTEN*. The panel was designed using the Ion Ampliseq Designer v.3.6, and it covers 42,320 bp of human genome sequences. The detailed methodology for the preparation of libraries of genomic DNA fragments, clonal emulsion PCR, sequencing and bioinformatic analysis of the results was described in our previously published paper [70].

### 4.7. Generation of miRNAs Expression Constructs

To ectopically express miR-129-5p and miR-3613-3p in cancer cell line SNU-1, HCT116, MCF-7, SKBR3, 769-P and A549, their DNA sequences were cloned into expression plasmids pSecTag2B (Invitrogen) by using standard genetic engineering techniques [72]. A full-length cDNA of miRNAs miR-129-5p (GGATCTTTTTGCGGTCTGGGCTTGCTGTTCCTCTCAACAGTAGTCAGGAAGCCCTTACCCCAAAAAGTATCT) and miR-3613-3p (TGGTTGGGTTTGGATTGTTGTACTTTTTTTTTTGTTCGTTGCATTTTTAGGAACAAAAAAAAAAGCCCAACCCTTCACACCACTTCA) were amplified using the Phusion DNA Polymerase (Thermo Scientific) with a pair of target-specific PCR primers (Table 8) containing the CciNI and NotI restriction sites (shown in italics). The amplification reaction was carried out with the following procedures: initial denaturation at 98 °C for 50 s followed by 10 consecutive cycles of denaturation at 98 °C for 10 s, annealing at 66 °C for 15 s, extension at 72 °C for 10 s and 20 cycles of 98 °C for 10 s, 72 °C for 15 s, and final extension at 70 °C for 5 min.

The amplification reaction was carried out with the following procedures: initial denaturation at 98 °C for 50 s followed by 10 consecutive cycles of denaturation at 98 °C for 10 s, annealing at 66 °C for 15 s, extension at 72 °C for 10 s and 20 cycles of 98 °C for 10 s, 72 °C for 15 s, and final extension at 70 °C for 5 min. The amplified miR-129-5p and miR-3613-3p were gel-purified by the CleanupMini kit (Evrogen, Moscow, Russia). After digestion with CciNI (SibEnzyme) and NotI (Thermo Scientific) restriction enzymes, the purified products were ligated into the cloning site of pSecTag2B vectors using T4 DNA ligase (Thermo scientific). Transfection of the ligase mixture was performed by the calcium transformation method. Competent cells of the *E. coli* XL1-Blue strain were used as transformants. Cell selection was carried out on Petri dishes with LB agar containing ampicillin. Colony screening was performed by PCR using standard vector primers along the length of the amplification products. Plasmid DNA was isolated from selected clones using a Plasmid Miniprep Color kit (Evrogen); the concentration was measured and analyzed by sequencing on an ABI310 genetic analyzer (Applied Biosystems, USA).

### 4.8. Transfection with miR-129-5p and miR-3613-3p Expression Plasmid

For transfection experiments, cancer cells SNU-1, HCT116, MCF-7, SKBR3, 769-P and A549 were seeded in 6-well plates with a density of 4 × 10^5^ cells/well (SNU-1) or 2.4×105 cells/well (all other cell lines) in complete growth medium and allowed to attach overnight. Cell number was estimated by staining with trypan blue and counting viable cells in a Neubauer chamber. Cells were transfected with 2000 ng miR-129-5p or miR-3613-3p expressing vector pSecTag2B or the empty plasmid as a mock control using TurboFect Transfection Reagent (Thermo Scientific) according to the manufacturer’s protocol. Cells were harvested at 24, 48, and 72 h following transfection, and the best efficiency was observed after 48 h. The transfection efficiency was verified using Western blotting, according to the previously published protocol [73]. The experiment was repeated at least three times with similar results.

### 4.9. Statistical Analysis

Statistical analysis of the results was performed by using the Statistica13.1 (StatSoft, Tulsa, OK, USA). The normality of sample distribution was evaluated using the Shapiro–Wilk test (for sample sizes < 50 samples) or the Kolmogorov–Smirnov test (for sample sizes > 50 samples). In the normal distribution, the mean and standard deviation were used. When the distribution was not normal, quantitative characteristics were described by using the median (Me) and the lower and upper quartiles (Q1–Q3). The comparison of the two groups by a quantitative indicator having a normal distribution was performed using the Student’s *t*-test; the comparison of the two groups’ variables with the distribution differed from the normal was performed by the Mann–Whitney test. The Kruskal–Wallis test was used when comparing three or more groups of variables, the distribution of which differed from the normal.

The direction and strength of the linear association between two variables were estimated using Spearman’s rank correlation coefficient (with a distribution of variables other than normal). Measurement of the correlations between *ARID1A* and miRNAs miR-129-5p and miR-3613-3p were performed using the RNAhybrid 2.2 tool.

A predictive model characterizing the dependence of a variable on factors was developed using linear regression. The Kaplan–Meier method was used to analyze overall survival. To explore the potential of microRNAs as a predictive biomarker, receiver operating characteristic curves (ROC-curves) were constructed, and the area under the curve (AUC) was calculated by calculating sensitivity and specificity at various threshold levels. Potentially useful predictors have been included in one-dimensional and multidimensional logistic regression analysis.

*p* < 0.05 values were considered statistically significant.

## 5. Conclusions

In our work, we have shown statistically significant differences between the expression of *ARID1A* and miR-129-5p in tissues of GC patients compared to sectional gastric samples of a healthy control group. MiR-129-5p was also differentially expressed in the plasma of GC patients. Expression of *ARID1A*, miR-129-5p and miR-3613-3p was associated with clinical and pathological characteristics of tumor growth, microsatellite instability (MSI) and mutations in *CDH1* and *TP53*. We performed an in vitro validation of predicted target pairs miR-129-5p/*ARID1A* and miR-3613-3p/*ARID1A* in cancer cell lines. The obtained data suggest a complex role of *ARID1A* and miRNAs miR-129-5p and miR-3613-3p in gastric tumorigenesis; however, additional studies are needed to confirm their potential clinical value.

## Figures and Tables

**Table 1:** Association of *ARID1A*, miR-129-5p and miR-3613-3p in GC tumor tissues with clinical and pathological characteristics of the patients.

Variables	Patients (N = 110)	*ARID1A* Expression			miR-129-5p Expression			miR-3613-3p Expression		
		M	Q1/Q3	*p*	M	Q1/Q3	*p*	M	Q1/Q3	*p*
Gender				0.4			0.6			0.2
Female	49	0.006	0.002/0.018		0.3	0.1/3.0		0.10	0.05/0.30	
Male	61	0.005	0.001/0.019		0.4	0.1/1.8		0.06	0.03/0.28	
Age (years)				0.2			0.076			0.05
<49	13	0.005	0.001/0.007		2.1	0.4/4.7		0.38	0.07/1.09	
>=50	97	0.006	0.001/0.019		0.4	0.1/1.8		0.06	0.03/0.24	
T				0.037 *			0.18			0.17
T1 + 2	53	0.007	0.003/0.019		0.25	0.1/1.4				
T3 + 4	57	0.003	0.001/0.016		0.64	0.1/3.0				
N				0.6			0.64			0.5
N0	60	0.006	0.000/0.019		0.42	0.1/1.8		0.06	0.03/0.26	
N0 + 1 + 3	50	0.005	0.001/0.018		0.51	0.1/3.0		0.13	0.03/0.35	
M				0.13			0.8			0.7
M0	101	0.006	0.001/0.019		0.4	0.1/2.3		0.07	0.03/0.28	
M1	9	0.003	0.001/0.005		0.6	0.2/1.7		0.18	0.01/0.31	
Stage				0.06			0.65			0.4
I + II	62	0.007	0.002/0.019		0.39	0.1/1.8		0.06	0.03/0.20	
III + IV	48	0.004	0.001/0.015		0.54	0.1/3.0		0.11	0.02/0.40	
Survival status				0.038 *			0.017 *			0.027 *
Alive	73	0.007	0.002/0.019		0.2	0.1/1.8		0.06	0.03/0.17	
Dead	37	0.003	0.001/0.013		1.2	0.3/3.3		0.23	0.04/0.49	
Lauren classification				0.001 *			0.2			0.011 *
Diffuse	81	0.007	0.002/0.020		0.2	0.1/1.9		0.06	0.02/0.18	
Intestinal	29	0.001	0.000/0.007		0.9	0.3/2.7		0.20	0.06/0.62	
Signet ring cells				0.044 *			0.7			0.3
No	71	0.006	0.001/0.014		0.4	0.1/2.6		0.07	0.03/0.31	
Yes	39	0.015	0.002/0.025		0.4	0.1/1.4		0.06	0.02/0.20	
Tumor localization				0.3			0.2			0.5
Antral region	20	0.006	0.003/0.013		1.0	0.3/1.4		0.08	0.05/0.24	
Body	73	0.006	0.001/0.018		0.6	0.1/3.0		0.07	0.03/0.31	
Cardia	17	0.006	0.003/0.020		0.2	0.1/0.2		0.06	0.02/0.09	
MSI (N = 57)				0.12			0.4			0.3
MSS	51	0.003	0.001/0.006		1.0	0.3/3.3		0.17	0.05/0.40	
H&L	6	0.010	0.009/0.017		2.2	1.6/2.9		0.40	0.20/0.59	
*TP53* (N = 50)				0.2			0.8			0.8
No	38	0.003	0.001/0.011		1.1	0.2/2.7		0.17	0.05/0.39	
Yes	12	0.007	0.004/0.011		0.6	0.3/1.6		0.11	0.05/0.32	
*CDH1* (N = 50)				0.04 *			0.7			0.4
No	45	0.003	0.001/0.010		0.6	0.2/2.8		0.17	0.05/0.42	
Yes	5	0.013	0.007/0.019		0.5	0.3/1.5		0.08	0.04/0.17	

* Statistically significant *p* < 0.05; N—number of patients in the study; M—median; Q1—quartile 25%; Q3—quartile 75%; *p*-value. Statistically significant p-values are indicated with an asterisk.

**Table 2:** Association of *ARID1A*, miR-129-5p and miR-3613-3p in GC adjacent non-tumor tissues with clinical and pathological characteristics of the patients.

Variables	Patients (N = 110)	*ARID1A* Expression			miR-129-5p Expression			miR-3613-3p Expression		
		M	Q1/Q3	*p*	M	Q1/Q3	*p*	M	Q1/Q3	*p*
Gender				0.7			0.6			0.5
Female	49	0.005	0.003/0.014		0.34	0.05/2.24		0.07	0.03/0.30	
Male	61	0.007	0.001/0.012		0.4	0.11/3.09		0.05	0.02/0.25	
Age (years)				0.075			0.2			0.011 *
<49	13	0.004	0.001/0.005		0.91	0.23/3.65		0.2	0.06/1.29	
>=50	97	0.007	0.002/0.015		0.34	0.07/2.24		0.05	0.02/0.19	
T				0.88			0.05			0.021 *
T1 + 2	53	0.006	0.001/0.012		0.27	0.05/1.0		0.04	0.02/0.10	
T3 + 4	57	0.007	0.001/0.016		0.91	0.1/3.8		0.07	0.02/0.77	
N				0.23			0.1			0.4
N0	60	0.005	0.001/0.010		0.45	0.13/2.2		0.05	0.02/0.16	
N1–3	50	0.008	0.002/0.017		0.22	0.1/3.3		0.06	0.03/0.68	
M				0.6			0.5			0.5
M0	101	0.006	0.001/0.014		0.40	0.08/2.47		0.05	0.02/0.23	
M1	9	0.006	0.004/0.008		0.26	0.17/4.43		0.07	0.05/0.68	
Stage				0.075			0.24			0.24
I + II	62	0.004	0.001/0.010		0.36	0.05/2.2		0.05	0.02/0.16	
III + IV	48	0.008	0.003/0.017		0.50	0.1/3.8		0.06	0.02/0.68	
Survival status				0.3			0.035 *			0.024 *
Alive	73	0.007	0.002/0.015		0.22	0.04/2.22		0.03	0.02/0.13	
Dead	37	0.005	0.001/0.011		0.61	0.24/3.54		0.10	0.05/0.55	
Lauren classification				0.002 *			0.059			0.2
Diffuse	81	0.008	0.004/0.016		0.22	0.06/2.16		0.05	0.02. 0.13	
Intestinal	29	0.003	0.001/0.006		1.25	0.27/4.38		0.14	0.03. 0.87	
Signet ring cells				0.3			0.2			0.99
No	71	0.005	0.002/0.011		0.63	0.09/3.34		0.05	0.02/0.51	
Yes	39	0.008	0.001/0.017		0.22	0.09/0.91		0.06	0.03/0.19	
Tumor localization				0.2			0.3			0.5
Antral region	20	0.004	0.001/0.011		0.33	0.09/1.21		0.03	0.02/0.07	
Body	73	0.006	0.001/0.012		0.54	0.09/3.34		0.07	0.03/0.51	
Cardia	17	0.008	0.005/0.022		0.31	0.18/2.82		0.06	0.03/0.09	
MSI (N = 57)				0.99			0.023 *			0.018 *
MSS	51	0.003	0.001/0.008		0.61	0.15/3.13		0.07	0.03/0.51	
H&L	6	0.002	0.001/0.009		5.25	3.87/5.94		1.08	0.77/1.63	
*TP53* (N = 50)				0.038 *			0.5			0.6
No	38	0.002	0.001/0.006		0.54	0.17/2.26		0.07	0.03/0.44	
Yes	12	0.008	0.004/0.011		1.40	0.27/4.56		0.17	0.03/0.89	
*CDH1* (N = 50)				0.7			0.3			0.5
No	45	0.003	0.001/0.008		0.61	0.16/3.2		0.07	0.03/0.50	
Yes	5	0.004	0.001/0.016		0.91	0.54/5.52		0.22	0.04/1.48	

* Statistically significant *p* < 0.05; N—number of patients in the study; M—median; Q1—quartile 25%; Q3—quartile 75%; *p*-value. Statistically significant p-values are indicated with an asterisk.

**Table 3:** Association of miR-129-5p and miR-3613-3p expression in plasma with clinical and pathological characteristics of GC patients.

Variables	Patients (N = 65)	miR-129-5p Expression			miR-3613-3p Expression		
		M	Q1/Q3	*p*	M	Q1/Q3	*p*
Gender				0.5			0.5
Female	29	0.02	0.01/0.05		0.0002	0.0001/0.0005	
Male	36	0.02	0.02/0.03		0.0003	0.0001/0.0006	
Age (years)				0.5			0.7
<49	9	0.02	0.01/0.05		0.0005	0.0001/0.0011	
>=50	56	0.02	0.02/0.03		0.0002	0.0001/0.0005	
T				0.005 *			0.17
T1 + 2	27	0.016	0.01/0.03		0.0002	0.0001/0.0005	
T3 + 4	38	0.026	0.02/0.05		0.0003	0.0001/0.0007	
N				0.005 *			0.11
N0	32	0.018	0.01/0.03		0.0002	0.0001/0.0005	
N1–3	33	0.028	0.02/0.05		0.0004	0.0001/0.0011	
M				0.004 *			0.3
M0	52	0.02	0.01/0.03		0.0002	0.0001/0.0005	
M1	13	0.05	0.02/0.13		0.0004	0.0001/0.0011	
Stage				0.006 *			0.05
I + II	31	0.018	0.01/0.03		0.0002	0.0001/0.0005	
III + IV	34	0.026	0.02/0.06		0.0004	0.0001/0.0011	
Survival status (N = 63)				0.8			0.8
Alive	61	0.02	0.01/0.04		0.0002	0.0001/0.0005	
Dead	2	0.02	0.01/0.02		0.0004	0.0002/0.0006	
Lauren classification (N = 58)				0.11			0.99
Diffuse	51	0.02	0.01/0.03		0.0002	0.0001/0.0005	
Intestinal	7	0.03	0.02/0.09		0.0002	0.0001/0.0007	
Signet ring cells				0.2			0.3
Yes	24	0.02	0.01/0.03		0.0002	0.0001/0.0005	
No	16	0.03	0.02/0.04		0.0003	0.0001/0.0010	
Tumor localization				0.43			0.71
(N = 61)							
Antral region	6	0.02	0.02/0.13		0.0002	0.0001/0.0014	
Cardia	10	0.03	0.02/0.04		0.0002	0.0001/0.0006	
Body	45	0.02	0.01/0.03		0.0003	0.0001/0.0005	

* Statistically significant *p* < 0.05; N—number of patients in the study; M—median; Q1—quartile 25%; Q3—quartile 75%; *p*-value. Statistically significant *p*-values are indicated with an asterisk.

**Table 4:** Characteristics of miR-129-5p and miR-3613-3p diagnostic value in plasma of GC patients.

MiRNAs	Variables	AUC	Specificity	Sensitivity
Combination	T	0.67	0.60	0.66
miR-129-5p		0.71	0.79	0.54
miR-3613-3p		0.59	0.51	0.67
Combination	N	0.67	0.45	0.80
miR-129-5p		0.71	0.54	0.79
miR-3613-3p		0.60	0.37	0.87
Combination	M	0.69	0.53	0.87
miR-129-5p		0.74	0.61	0.89
miR-3613-3p		0.52	0.31	0.81
Combination	Stage	0.69	0.50	0.78
miR-129-5p		0.71	0.55	0.74
miR-3613-3p		0.64	0.52	0.74

**Table 5:** The clinical and pathological characteristics of patients.

Variables	GC Tissues		Plasma	
	Patients (N = 110)	Healthy Controls (N = 38)	Patients (N = 65)	Healthy Controls (N = 48)
Gender				
Female	49 (44.5%)	14 (37%)	29 (45%)	28 (58%)
Male	61 (55.5%)	24 (63%)	36 (55%)	20 (42%)
Age (years)				
<49	13 (12%)	17 (45%)	9 (14%)	16 (33%)
>=50	97 (88%)	21 (55%)	56 (86%)	32 (67%)
T				
T1	13 (12%)		3 (5%)	
T2	40 (36%)		24 (37%)	
T3	30 (27%)		29 (44%)	
T4	27 (25%)		9 (14%)	
N				
N0	60 (54%)		32 (49%)	
N1	35 (32%)		27 (42%)	
N2	14 (13%)		4 (6%)	
N3	1 (1%)		2 (3%)	
M				
M0	101 (92%)		52 (80%)	
M1	9 (8%)		13 (20%)	
Stage				
I	20 (18%)		3 (5%)	
II	42 (38%)		28 (43%)	
III	41 (37%)		21 (32%)	
IV	7 (7%)		13 (20%)	
Survival status			(N = 63)	
Alive	73 (66%)		61 (97%)	
Dead	37 (34%)		2 (3%)	
Lauren classification			(N = 58)	
Diffuse	81 (74%)		51 (88%)	
Intestinal	29 (26%)		7 (12%)	
Signet ring cells				
No	71 (65%)		43 (66%)	
Yes	39 (35%)		22 (34%)	
Tumor localization				
Antral region	20 (18%)		6 (10%)	
Body	73 (66%)		40 (61%)	
Cardia	17 (16%)		19 (29%)	

N—Number of cases (%).

**Table 6:** Primer sequences.

Primer	Sequence
*ARID1A*_F	5′-CAGTACCTGCCTCGCACATA-3′
*ARID1A*_R	5′- GCCAGGAGACCAGACTTGAG-3′
*GAPDH*_F	5′-CACCCACTCCTCCACCTTTG-3′
*GAPDH*_R	5′-CCACCACCCTGTTGCTGTAG-3′
miR-129-5p	5′- TTTTGCGGTCTGGGCTTGC-3′
miR-3613-3p	5′-ACAAAAAAAAAAGCCCAACCCTTC-3′
RNU6B	5′-TGCGCAAGGATGACACGCAA-3′

**Table 7:** Markers for the MSI analysis and their conditions.

Marker	Locus	Genome Coordinates	Primers	Length (nt)
NR21	14q11.2	14:23,125,294– 23,183,659	FAM–GTCGCTGGCACAGTTCTA R–CTGGTCACTCGCGTTTACAA	110
NR24	2q11.1	2:95,165,808– 95,184,316	FAM–CTGAATTTTACCTCCTGAC R–ATTGTGCCATTGCATTCCAA	129
BAT25	4q12	4:54,657,927– 54,740,714	FAM–TCGCCTCCAAGAATGTAAGT R–TCTGCATTTTAACTATGGCTC	124
BAT26	2p21–p16	2:47,403,066– 47,634,500	FAM–TGACTACTTTTGACTTCAGCC R–AACCATTCAACATTTTTAACCC	122
NR27	2p22.1	2:39,248,940– 39,437,311	FAM–AACCATGCTTGCAAACCACT R–CGATAATACTAGCAATGACC	90

**Table 8:** Primers for amplification of DNA sequences of miRNAs.

Primer	Sequence	Length of Amplicon (nt)
miR-129 F	ATAT***GCTAGC***GGATCTTTTTGCGGTCTGGGCTTGC	92
miR-129 R	AT***GCGGCCGC***AGATACTTTTTGGGGTAAGGGCTTCCTG	
miR-3613 F	ATAT***GCTAGC***TGGTTGGGTTTGGATTGTTGTACTT	107
miR-3613 R	AT***GCGGCCGC***TGAAGTGGTGTGAAGGGTTGG	

## Data Availability

Dataset available on request from the authors.

## References

[1] Shimizu D., Kanda M., Kodera Y. (2018). Review of Recent Molecular Landscape Knowledge of Gastric Cancer. Histol. Histopathol..

[2] Szelenberger R., Kacprzak M., Saluk-Bijak J., Zielinska M., Bijak M. (2019). Plasma MicroRNA as a Novel Diagnostic. Clin. Chim. Acta.

[3] Aalami A.H., Aalami F., Sahebkar A. (2023). Gastric Cancer and Circulating MicroRNAs: An Updated Systematic Review and Diagnostic Meta-Analysis. Curr. Med. Chem..

[4] Matsuzaki J., Ochiya T. (2017). Circulating MicroRNAs and Extracellular Vesicles as Potential Cancer Biomarkers: A Systematic Review. Int. J. Clin. Oncol..

[5] O’Brien J., Hayder H., Zayed Y., Peng C. (2018). Overview of MicroRNA Biogenesis, Mechanisms of Actions, and Circulation. Front. Endocrinol..

[6] Piletič K., Kunej T. (2016). MicroRNA Epigenetic Signatures in Human Disease. Arch. Toxicol..

[7] Bure I.V., Mikhaylenko D.S., Kuznetsova E.B., Alekseeva E.A., Bondareva K.I., Kalinkin A.I., Lukashev A.N., Tarasov V.V., Zamyatnin A.A., Nemtsova M.V. (2020). Analysis of MiRNA Expression in Patients with Rheumatoid Arthritis during Olokizumab Treatment. J. Pers. Med..

[8] Khoodoruth M.A.S., Khoodoruth W.N.C.-K., Uroos M., Al-Abdulla M., Khan Y.S., Mohammad F. (2024). Diagnostic and Mechanistic Roles of MicroRNAs in Neurodevelopmental & Neurodegenerative Disorders. Neurobiol. Dis..

[9] Goel H., Goel A. (2024). MicroRNA and Rare Human Diseases. Genes.

[10] Chakrabortty A., Patton D.J., Smith B.F., Agarwal P. (2023). MiRNAs: Potential as Biomarkers and Therapeutic Targets for Cancer. Genes.

[11] Meng R., Fang J., Yu Y., Hou L.K., Chi J.R., Chen A.X., Zhao Y., Cao X.C. (2018). MiR-129-5p Suppresses Breast Cancer Proliferation by Targeting CBX4. Neoplasma.

[12] Wu Q., Meng W.-Y., Jie Y., Zhao H. (2018). LncRNA MALAT1 Induces Colon Cancer Development by Regulating MiR-129-5p/HMGB1 Axis. J. Cell. Physiol..

[13] Liu K., Huang J., Ni J., Song D., Ding M., Wang J., Huang X., Li W. (2017). MALAT1 Promotes Osteosarcoma Development by Regulation of HMGB1 via MiR-142-3p and MiR-129-5p. Cell Cycle.

[14] Zhang Y., An J., Lv W., Lou T., Liu Y., Kang W. (2016). MiRNA-129-5p Suppresses Cell Proliferation and Invasion in Lung Cancer by Targeting Microspherule Protein 1, E-Cadherin and Vimentin. Oncol. Lett..

[15] Yu X., Song H., Xia T., Han S., Xiao B., Luo L., Xi Y., Guo J. (2013). Growth Inhibitory Effects of Three MiR-129 Family Members on Gastric Cancer. Gene.

[16] Wang Q., Yu J. (2018). MiR-129-5p Suppresses Gastric Cancer Cell Invasion and Proliferation by Inhibiting COL1A1. Biochem. Cell Biol..

[17] Xiang F., Xu X. (2022). CirRNA F-CircEA-2a Suppresses the Role of MiR-3613-3p in Colorectal Cancer by Direct Sponging and Predicts Poor Survival. Cancer Manag. Res..

[18] Bibi F., Naseer M.I., Alvi S.A., Yasir M., Jiman-Fatani A.A., Sawan A., Abuzenadah A.M., Al-Qahtani M.H., Azhar E.I. (2016). MicroRNA Analysis of Gastric Cancer Patients from Saudi Arabian Population. BMC Genom..

[19] Chen C., Pan Y., Bai L., Chen H., Duan Z., Si Q., Zhu R., Chuang T.-H., Luo Y. (2021). MicroRNA-3613-3p Functions as a Tumor Suppressor and Represents a Novel Therapeutic Target in Breast Cancer. Breast Cancer Res..

[20] Zhang D., Liu E., Kang J., Yang X., Liu H. (2017). MiR-3613-3p Affects Cell Proliferation and Cell Cycle in Hepatocellular Carcinoma. Oncotarget.

[21] Castro-Magdonel B.E., Orjuela M., Alvarez-Suarez D.E., Camacho J., Cabrera-Muñoz L., Sadowinski-Pine S., Medina-Sanson A., Lara-Molina C., García-Vega D., Vázquez Y. (2020). Circulating MiRNome Detection Analysis Reveals 537 MiRNAS in Plasma, 625 in Extracellular Vesicles and a Discriminant Plasma Signature of 19 MiRNAs in Children with Retinoblastoma from Which 14 Are Also Detected in Corresponding Primary Tumors. PLoS ONE.

[22] Nowak I., Boratyn E., Durbas M., Horwacik I., Rokita H. (2018). Exogenous Expression of MiRNA-3613-3p Causes APAF1 Downregulation and Affects Several Proteins Involved in Apoptosis in BE(2)-C Human Neuroblastoma Cells. Int. J. Oncol..

[23] Slattery M.L., Herrick J.S., Mullany L.E., Wolff E., Hoffman M.D., Pellatt D.F., Stevens J.R., Wolff R.K. (2016). Colorectal Tumor Molecular Phenotype and MiRNA: Expression Profiles and Prognosis. Mod. Pathol..

[24] Ahsen M.E., Boren T.P., Singh N.K., Misganaw B., Mutch D.G., Moore K.N., Backes F.J., McCourt C.K., Lea J.S., Miller D.S. (2017). Sparse Feature Selection for Classification and Prediction of Metastasis in Endometrial Cancer. BMC Genom..

[25] Agarwal V., Bell G.W., Nam J.-W., Bartel D.P. (2015). Predicting Effective MicroRNA Target Sites in Mammalian MRNAs. eLife.

[26] Tokar T., Pastrello C., Rossos A.E.M., Abovsky M., Hauschild A.-C., Tsay M., Lu R., Jurisica I. (2018). MirDIP 4.1-Integrative Database of Human MicroRNA Target Predictions. Nucleic Acids Res..

[27] Huang J., Zhao Y.-L., Li Y., Fletcher J.A., Xiao S. (2007). Genomic and Functional Evidence for an ARID1A Tumor Suppressor Role. Genes Chromosomes Cancer.

[28] Wilson B.G., Roberts C.W.M. (2011). SWI/SNF Nucleosome Remodellers and Cancer. Nat. Rev. Cancer.

[29] Barisic D., Chin C.R., Meydan C., Teater M., Tsialta I., Mlynarczyk C., Chadburn A., Wang X., Sarkozy M., Xia M. (2024). ARID1A Orchestrates SWI/SNF-Mediated Sequential Binding of Transcription Factors with ARID1A Loss Driving Pre-Memory B Cell Fate and Lymphomagenesis. Cancer Cell.

[30] Wu J.N., Roberts C.W.M. (2013). *ARID1A* Mutations in Cancer: Another Epigenetic Tumor Suppressor?. Cancer Discov..

[31] Yamamoto H., Watanabe Y., Maehata T., Morita R., Yoshida Y., Oikawa R., Ishigooka S., Ozawa S.-I., Matsuo Y., Hosoya K. (2014). An Updated Review of Gastric Cancer in the Next-Generation Sequencing Era: Insights from Bench to Bedside and Vice Versa. World J. Gastroenterol..

[32] Zhang B., Pan X., Cobb G.P., Anderson T.A. (2007). MicroRNAs as Oncogenes and Tumor Suppressors. Dev. Biol..

[33] Blandino G., Fazi F., Donzelli S., Kedmi M., Sas-Chen A., Muti P., Strano S., Yarden Y. (2014). Tumor Suppressor MicroRNAs: A Novel Non-Coding Alliance against Cancer. FEBS Lett..

[34] Farazi T.A., Spitzer J.I., Morozov P., Tuschl T. (2011). MiRNAs in Human Cancer. J. Pathol..

[35] Wang S., Chen Y., Yu X., Lu Y., Wang H., Wu F., Teng L. (2019). MiR-129-5p Attenuates Cell Proliferation and Epithelial Mesenchymal Transition via HMGB1 in Gastric Cancer. Pathol. Res. Pract..

[36] Yan L., Sun K., Liu Y., Liang J., Cai K., Gui J. (2017). MiR-129-5p Influences the Progression of Gastric Cancer Cells through Interacting with SPOCK1. Tumour. Biol..

[37] He J., Ge Q., Lin Z., Shen W., Lin R., Wu J., Wang B., Lu Y., Chen L., Liu X. (2020). MiR-129-5p Induces Cell Cycle Arrest through Modulating HOXC10/Cyclin D1 to Inhibit Gastric Cancer Progression. FASEB J..

[38] Jiang Z., Wang H., Li Y., Hou Z., Ma N., Chen W., Zong Z., Chen S. (2016). MiR-129-5p Is down-Regulated and Involved in Migration and Invasion of Gastric Cancer Cells by Targeting Interleukin-8. Neoplasma.

[39] Liu Z., Sun J., Wang X., Cao Z. (2021). MicroRNA-129-5p Promotes Proliferation and Metastasis of Hepatocellular Carcinoma by Regulating the BMP2 Gene. Exp. Ther. Med..

[40] Liu Y., Yang Y., Du J., Lin D., Li F. (2020). MiR-3613-3p from Carcinoma-Associated Fibroblasts Exosomes Promoted Breast Cancer Cell Proliferation and Metastasis by Regulating SOCS2 Expression. IUBMB Life.

[41] Pu Q., Huang Y., Lu Y., Peng Y., Zhang J., Feng G., Wang C., Liu L., Dai Y. (2016). Tissue-Specific and Plasma MicroRNA Profiles Could Be Promising Biomarkers of Histological Classification and TNM Stage in Non-Small Cell Lung Cancer. Thorac. Cancer.

[42] Schallenberg S., Bork J., Essakly A., Alakus H., Buettner R., Hillmer A.M., Bruns C., Schroeder W., Zander T., Loeser H. (2020). Loss of the SWI/SNF-ATPase Subunit Members SMARCF1 (ARID1A), SMARCA2 (BRM), SMARCA4 (BRG1) and SMARCB1 (INI1) in Oesophageal Adenocarcinoma. BMC Cancer.

[43] Qadir J., Majid S., Khan M.S., Rashid F., Wani M.D., Din I., Bashir H. (2020). AT-Rich Interaction Domain 1A Gene Variations: Genetic Associations and Susceptibility to Gastric Cancer Risk. Pathol. Oncol. Res..

[44] Xu S., Tang C. (2021). The Role of ARID1A in Tumors: Tumor Initiation or Tumor Suppression?. Front. Oncol..

[45] Tober J.M., Halske C., Behrens H.-M., Krüger S., Röcken C. (2019). Intratumoral Heterogeneity and Loss of ARID1A Expression in Gastric Cancer Correlates with Increased PD-L1 Expression in Western Patients. Hum. Pathol..

[46] Fontana B., Gallerani G., Salamon I., Pace I., Roncarati R., Ferracin M. (2023). ARID1A in Cancer: Friend or Foe?. Front. Oncol..

[47] Ibarrola-Villava M., Llorca-Cardeñosa M.J., Tarazona N., Mongort C., Fleitas T., Perez-Fidalgo J.A., Roselló S., Navarro S., Ribas G., Cervantes A. (2015). Deregulation of ARID1A, CDH1, CMET and PIK3CA and Target-Related MicroRNA Expression in Gastric Cancer. Oncotarget.

[48] Wang D., Chen Y., Pan K., Wang W., Chen S., Chen J., Zhao J., Lv L., Pan Q., Li Y. (2012). Decreased Expression of the ARID1A Gene Is Associated with Poor Prognosis in Primary Gastric Cancer. PLoS ONE.

[49] Inada R., Sekine S., Taniguchi H., Tsuda H., Katai H., Fujiwara T., Kushima R. (2015). ARID1A Expression in Gastric Adenocarcinoma: Clinicopathological Significance and Correlation with DNA Mismatch Repair Status. World J. Gastroenterol..

[50] Han N., Kim M.A., Lee H.S., Kim W.H. (2016). Loss of ARID1A Expression Is Related to Gastric Cancer Progression, Epstein-Barr Virus Infection, and Mismatch Repair Deficiency. Appl. Immunohistochem. Mol. Morphol..

[51] Sun X., Wang S.C., Wei Y., Luo X., Jia Y., Li L., Gopal P., Zhu M., Nassour I., Chuang J.-C. (2017). Arid1a Has Context-Dependent Oncogenic and Tumor Suppressor Functions in Liver Cancer. Cancer Cell.

[52] Qadir J., Majid S., Khan M.S., Rashid F., Wani M.D., Bhat S.A. (2021). Implication of ARID1A Undercurrents and PDL1, TP53 Overexpression in Advanced Gastric Cancer. Pathol. Oncol. Res..

[53] Kim M.J., Gu M.J., Chang H.-K., Yu E. (2015). Loss of ARID1A Expression Is Associated with Poor Prognosis in Small Intestinal Carcinoma. Histopathology.

[54] Kim Y.-S., Jeong H., Choi J.-W., Oh H.E., Lee J.-H. (2017). Unique Characteristics of ARID1A Mutation and Protein Level in Gastric and Colorectal Cancer: A Meta-Analysis. Saudi J. Gastroenterol..

[55] Wang X., Che K., Shi T., Liu Q., Xu X., Wu H., Yu L., Liu B., Wei J. (2022). Loss of ARID1A Expression Is Associated with Systemic Inflammation Markers and Has Important Prognostic Significance in Gastric Cancer. J. Cancer Res. Clin. Oncol..

[56] Sakuratani T., Takeuchi T., Yasufuku I., Iwata Y., Saigo C., Kito Y., Yoshida K. (2021). Downregulation of ARID1A in Gastric Cancer Cells: A Putative Protective Molecular Mechanism against the Harakiri-Mediated Apoptosis Pathway. Virchows Arch..

[57] Saito M., Kohno T., Kono K. (2020). Heterogeneity of ARID1A Expression in Gastric Cancer May Affect Patient Survival and Therapeutic Efficacy. Hum. Pathol..

[58] Sasaki T., Kohashi K., Kawatoko S., Ihara E., Oki E., Nakamura M., Ogawa Y., Oda Y. (2022). Tumor Progression by Epithelial-Mesenchymal Transition in ARID1A- and SMARCA4-Aberrant Solid-Type Poorly Differentiated Gastric Adenocarcinoma. Virchows Arch..

[59] Ashizawa M., Saito M., Min A.K.T., Ujiie D., Saito K., Sato T., Kikuchi T., Okayama H., Fujita S., Endo H. (2019). Prognostic Role of ARID1A Negative Expression in Gastric Cancer. Sci. Rep..

[60] Yan H.-B., Wang X.-F., Zhang Q., Tang Z.-Q., Jiang Y.-H., Fan H.-Z., Sun Y., Yang P.-Y., Liu F. (2014). Reduced Expression of the Chromatin Remodeling Gene ARID1A Enhances Gastric Cancer Cell Migration and Invasion via Downregulation of E-Cadherin Transcription. Carcinogenesis.

[61] Wang K., Kan J., Yuen S.T., Shi S.T., Chu K.M., Law S., Chan T.L., Kan Z., Chan A.S.Y., Tsui W.Y. (2011). Exome Sequencing Identifies Frequent Mutation of ARID1A in Molecular Subtypes of Gastric Cancer. Nat. Genet..

[62] Allo G., Bernardini M.Q., Wu R.-C., Shih I.-M., Kalloger S., Pollett A., Gilks C.B., Clarke B.A. (2014). ARID1A Loss Correlates with Mismatch Repair Deficiency and Intact P53 Expression in High-Grade Endometrial Carcinomas. Mod. Pathol..

[63] Yang Y., Yin Z.X., Wang Z.Y., Tian S.B., Wang H.C., Zhang F.X., Li L.P., Zheng C., Kong S. (2020). MiR-7641 Depletion Suppresses Proliferation of Gastric Cancer Cells by Targeting ARID1A. Anticancer. Drugs.

[64] Zhu Y., Li K., Yan L., He Y., Wang L., Sheng L. (2020). MiR-223-3p Promotes Cell Proliferation and Invasion by Targeting *Arid1a* in Gastric Cancer. Acta Biochim. Biophys. Sin..

[65] Yang Y., Zhao X., Li H.-X. (2016). MiR-221 and MiR-222 Simultaneously Target ARID1A and Enhance Proliferation and Invasion of Cervical Cancer Cells. Eur. Rev. Med. Pharmacol. Sci..

[66] Xiao W., Lou N., Ruan H., Bao L., Xiong Z., Yuan C., Tong J., Xu G., Zhou Y., Qu Y. (2017). Mir-144-3p Promotes Cell Proliferation, Metastasis, Sunitinib Resistance in Clear Cell Renal Cell Carcinoma by Downregulating ARID1A. Cell Physiol. Biochem..

[67] Wang N., Zhou Y., Zheng L., Li H. (2014). MiR-31 Is an Independent Prognostic Factor and Functions as an Oncomir in Cervical Cancer via Targeting ARID1A. Gynecol. Oncol..

[68] Polyakova E.A., Zaraiskii M.I., Mikhaylov E.N., Baranova E.I., Galagudza M.M., Shlyakhto E.V. (2021). Association of Myocardial and Serum MiRNA Expression Patterns with the Presence and Extent of Coronary Artery Disease: A Cross-Sectional Study. Int. J. Cardiol..

[69] Shubin V., Shelygin Y., Achkasov S., Sushkov O., Nazarov I., Ponomarenko A., Alimova I., Loginova A., Tsukanov A. (2022). Microsatellite Instability in Russian Patients with Colorectal Cancer. Int. J. Mol. Sci..

[70] Nemtsova M.V., Kalinkin A.I., Kuznetsova E.B., Bure I.V., Alekseeva E.A., Bykov I.I., Khorobrykh T.V., Mikhaylenko D.S., Tanas A.S., Kutsev S.I. (2020). Clinical Relevance of Somatic Mutations in Main Driver Genes Detected in Gastric Cancer Patients by Next-Generation DNA Sequencing. Sci. Rep..

[71] Scarpa A., Sikora K., Fassan M., Rachiglio A.M., Cappellesso R., Antonello D., Amato E., Mafficini A., Lambiase M., Esposito C. (2013). Molecular Typing of Lung Adenocarcinoma on Cytological Samples Using a Multigene Next Generation Sequencing Panel. PLoS ONE.

[72] Green M.R., Sambrook J. (2021). A Guide to Cloning the Products of Polymerase Chain Reactions. Cold Spring Harb. Protoc..

[73] Rudzinska-Radecka M., Frolova A.S., Balakireva A.V., Gorokhovets N.V., Pokrovsky V.S., Sokolova D.V., Korolev D.O., Potoldykova N.V., Vinarov A.Z., Parodi A. (2022). In Silico, In Vitro, and Clinical Investigations of Cathepsin B and Stefin A MRNA Expression and a Correlation Analysis in Kidney Cancer. Cells.

